# The full-length transcriptome of *C. elegans* using direct RNA sequencing

**DOI:** 10.1101/gr.251314.119

**Published:** 2020-02

**Authors:** Nathan P. Roach, Norah Sadowski, Amelia F. Alessi, Winston Timp, James Taylor, John K. Kim

**Affiliations:** 1 Department of Biology, Johns Hopkins University, Baltimore, Maryland 21218, USA;; 2 Department of Biomedical Engineering, Department of Molecular Biology and Genetics, Johns Hopkins University, Baltimore, Maryland 21218, USA;; 3 Department of Computer Science, Johns Hopkins University, Baltimore, Maryland 21218, USA

## Abstract

Current transcriptome annotations have largely relied on short read lengths intrinsic to the most widely used high-throughput cDNA sequencing technologies. For example, in the annotation of the *Caenorhabditis elegans* transcriptome, more than half of the transcript isoforms lack full-length support and instead rely on inference from short reads that do not span the full length of the isoform. We applied nanopore-based direct RNA sequencing to characterize the developmental polyadenylated transcriptome of *C. elegans*. Taking advantage of long reads spanning the full length of mRNA transcripts, we provide support for 23,865 splice isoforms across 14,611 genes, without the need for computational reconstruction of gene models. Of the isoforms identified, 3452 are novel splice isoforms not present in the WormBase WS265 annotation. Furthermore, we identified 16,342 isoforms in the 3′ untranslated region (3′ UTR), 2640 of which are novel and do not fall within 10 bp of existing 3′-UTR data sets and annotations. Combining 3′ UTRs and splice isoforms, we identified 28,858 full-length transcript isoforms. We also determined that poly(A) tail lengths of transcripts vary across development, as do the strengths of previously reported correlations between poly(A) tail length and expression level, and poly(A) tail length and 3′-UTR length. Finally, we have formatted this data as a publicly accessible track hub, enabling researchers to explore this data set easily in a genome browser.

The nematode *Caenorhabditis elegans* is an ideal experimental model organism due to its compact, well-annotated genome (The *C. elegans* Sequencing Consortium 1998; Wilson 1999; Hillier et al. 2005; Gerstein et al. 2010), invariant cell lineage (Sulston et al. 1983), and wide array of molecular methods. Our current understanding of the *C. elegans* transcriptome has been determined with EST- and cDNA-based libraries, and Illumina-based cDNA and RNA sequencing (Walhout et al. 2000; Reboul et al. 2001; Lamesch et al. 2004; Hillier et al. 2009; Gerstein et al. 2010; Spieth et al. 2014; Tourasse et al. 2017). Most coding sequences (CDSs) span more than 600 nt (excluding introns), and the typical *C. elegans* gene contains 6.4 coding exons on average (Spieth et al. 2014).

3′ untranslated regions (3′ UTRs) are critically important features of mRNA transcripts that contain binding sites for RNA-binding proteins and small noncoding RNAs (Cai et al. 2009; Szostak and Gebauer 2013). Regulation of 3′-UTR length can therefore have profound impact on mRNA expression, stability, and localization (Kuersten and Goodwin 2003; Andreassi and Riccio 2009; Mayr and Bartel 2009). Large-scale sequencing of the *C. elegans* 3′ UTRs revealed median lengths of 130–140 nt, with an average length of ∼211 nt, although 3′-UTR length distributions have been shown to vary by cell and tissue type (e.g., oocytes have a median 3′-UTR length of ∼157 nt) (Mangone et al. 2010; Jan et al. 2011; West et al. 2018). In addition, poly(A) tails in *C. elegans* have a median length of ∼57 nt at the L4 stage, and short poly(A) tail lengths are a feature of highly expressed genes (Lima et al. 2017).

The average transcript in *C. elegans* is significantly longer than the maximum possible read length of Illumina sequencing. Therefore, current approaches to annotate the full-length structure of the average *C. elegans* transcript isoform rely on manual curation of gene models based on a variety of data types, while more generally computational approaches to assemble transcript structures from bulk, short-read sequencing data utilize computationally expensive and imperfect inference (Williams et al. 2011; Trapnell et al. 2012; Spieth et al. 2014; Pertea et al. 2015). Calculating poly(A) tail lengths requires a sequencing approach capable of resolving long homopolymers, and determining 3′-UTR structures requires an experimental or computational means of determining which reads reflect the 3′-most base included in the transcript before cleavage and polyadenylation. The specialized protocols and analyses used to measure poly(A) tail length and identify 3′ UTRs with short-read sequencing approaches cannot directly link these measurements to their splice isoform of origin and, in the case of 3′-UTR identification, instead rely on assigning putative cleavage sites to the nearest overlapping or upstream gene (Mangone et al. 2010; Jan et al. 2011; Chang et al. 2014; Subtelny et al. 2014; Blazie et al. 2017; Diag et al. 2018).

Nanopore sequencing, in contrast, has no theoretical upper limit to read length and is capable of sequencing transcripts from end to end at a single molecule level (Garalde et al. 2018; Jenjaroenpun et al. 2018; Workman et al. 2019). Nanopore-based sequencing methods have been used to annotate transcriptome structure in a variety of organisms ranging from the relatively simple *Saccharomyces cerevisiae* to complex human cell lines (Byrne et al. 2017; Bayega et al. 2018; Garalde et al. 2018; Jenjaroenpun et al. 2018; Tang et al. 2018; Volden et al. 2018; Kadobianskyi et al. 2019; Sessegolo et al. 2019; Workman et al. 2019). In nanopore-based direct RNA sequencing (dRNA-seq), RNA reads are captured by the 3′ end of their poly(A) tail and sequenced in the 3′ to 5′ direction natively, thus directly measuring the RNA molecule. The full length of the poly(A) tail is sequenced and, using a trained hidden Markov model, the length of the poly(A) tail for each read can be estimated (Workman et al. 2019). The 3′-most base in the alignment should reflect the true cleavage and polyadenylation site for the full transcript represented by that read, provided that base-calling, trimming of poly(A) and adapter sequences, and alignment had sufficient precision. Despite these advantages, adoption of dRNA-seq and other nanopore-based sequencing methods is hindered due to the technology's high error rates and the relative lack of bioinformatics tools and analysis pipelines designed for long, error-rich reads.

In this study, we have generated an atlas of postembryonic transcript structure using dRNA-seq to sequence RNA extracted from the major stages of the *C. elegans* developmental life cycle. We provide full-length support for both previously annotated and novel transcript splice isoforms. Furthermore, we identify and characterize 3′ UTRs and compare these to known 3′-UTR data sets. We also estimate poly(A) tail lengths for our reads and examine their length characteristics across development. Finally, we have made this data available both in raw formats and as a custom track hub.

## Results

### Collection and sequencing of developmentally staged *C. elegans*

**Figure 1.**
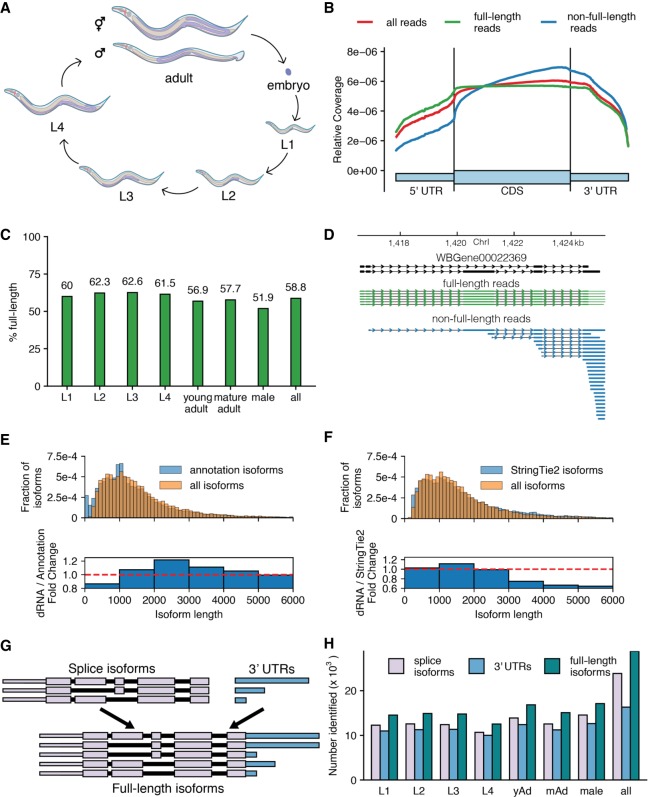
Overview of approach and sequencing of full-length isoforms. (*A*) Diagram of the *C. elegans* life cycle. (*B*) Plot of normalized coverage across the average coding gene with full-length (green), non-full-length (blue), and all reads (red) considered. (*C*) Percentage of reads that passed filtering and were called full-length in each stage. (*D*) Example locus showing reads aligning to the *WBGene00022369* locus (black). (*E*) Comparison of length distributions of isoforms present in the WormBase WS265 annotation and splice isoforms identified by this study displayed as a density plot (*top*) and as the fold change of the densities (*bottom*). (*F*) As in *E*, comparison of length distribution of isoforms assembled by StringTie2 using Illumina-based RNA-seq from across *C elegans* development and splice isoforms identified by this study. (*G*) Schematic defining “full-length isoform” as a combination of splice isoform and 3′-UTR isoform. (*H*) Number of splice, 3′-UTR, and full-length isoforms observed across all stages. (yAd) Young adult, (mAd) mature adult. Exact numbers can be found in Supplemental Table 3.

To capture the diversity of transcript isoforms expressed across *C. elegans* development, we created dRNA-seq libraries in technical duplicates from larval stages L1 to L4, as well as young and mature hermaphrodite adults (Fig. 1A; Corsi et al. 2015). Because wild-type *C. elegans* exists primarily as hermaphrodites with spontaneous males (<0.5%) emerging in the population through chromosome nondisjunction, we also obtained a male-enriched sample using a *him-8* mutant that disrupts X Chromosome segregation (Hodgkin et al. 1979; Broverman and Meneely 1994; Phillips et al. 2005). We further enriched for the male subpopulation by filtering them through a 35-µm mesh that allows the males to be collected in the filtrate.

Libraries were generated from RNA isolated by TRI Reagent (Ambion), poly(A)-selected, and prepared for sequencing following the Oxford Nanopore Technologies SQK-RNA001 kit protocol with the exception of using SuperScript IV (Thermo Fisher Scientific) in the optional reverse transcription step. The libraries were sequenced on an Oxford Nanopore Technologies GridION X5 (model #GRD-X5B002). Base-calling and adapter trimming of the reads was performed using poreplex (running albacore) (https://github.com/hyeshik/poreplex), resulting in over 540,000 reads that passed base-calling quality control for each developmental stage sequenced, and 5.54 million total reads (Supplemental Table 1). Reads had mean per base quality scores above 10 for each developmentally staged sample and median per base quality scores ranging between nine and 10 for each sample. Reads were aligned to the ce11 genome using minimap2, which successfully aligned 87.8% of our reads (Supplemental Table 2; Li 2018). Median read lengths ranged between 573 and 687 for a given sample, while average read lengths were significantly longer, ranging from 739 to 934. Note that nanopore sequencing reads are currently unable to capture the last 10–15 bases proximal to the 5′ end because of the structure of the pore-motor protein-RNA assembly as reported previously (Workman et al. 2019). The average percent reference identities of our alignments (as calculated by the NanoPack software suite [De Coster et al. 2018]) ranged between 85.3% and 86.9% depending on the sample, suggesting an error rate of ∼14%–15% in our data sets. This error rate and the loss of the 5′-most 10–15 bases prevented us from examining splice leader-based *trans* splicing, a common RNA modification in *C. elegans* (see discussion in Supplemental Material). The average length of unique splice isoforms identified in our sequencing libraries was 1596 nt (Fig. 1E,F and discussed below), which was consistent with the annotated average length of transcripts in the WormBase WS265 annotation of the *C. elegans* transcriptome (1574 nt) (Lee et al. 2018; WormBase web site [https://wormbase.org], release WS265 2018).

### Identifying reads representing full-length transcripts

While the majority of our reads correspond to full-length transcripts (Fig. 1B,C), a substantial fraction of aligned reads failed to span the full length of an annotated coding sequence (31.8% of the unfiltered genome aligned reads); these reads were predominantly truncated relative to annotated isoforms at their 5′ ends, resulting in a 3′ bias in coverage from our total reads (e.g., Fig. 1D). Including these reads in our downstream analysis would have artificially inflated the number of isoforms identified. Therefore, to make use of the long read lengths possible through dRNA-seq, reduce this 3′ bias, and eliminate the need to computationally reconstruct gene models, reads were filtered to ensure that only high-quality reads corresponding to full-length transcripts were considered (see Methods and Supplemental Fig. 1 for an outline of the entire analysis). Reads were considered full length in our filtering approach if they passed our complete filtering pipeline. Briefly, reads were discarded if they (1) contained large insertions or large 3′ softclips (i.e., bases at the end of a read that fail to align), (2) had no detectable poly(A) tail, (3) had 5′ ends that had insufficient evidence of corresponding to a transcription start site (TSS), (4) had a donor or acceptor splice site that could not be assigned to an annotated donor or acceptor splice site (i.e., a splice site not within 15 bp of an annotated splice site), (5) had retained introns, or (6) had 3′ ends that had insufficient evidence of corresponding to a bona fide polyadenylation site.

For 5′ end filtering criterion (step 3 above), we implemented a stringent 5′ filtering step that kept reads if their 5′ ends fell within −100 to +15 of an annotated transcription start site or were supported by 5′ SAGE data or high-throughput sequencing of RNA polymerase II initiation sites (Chen et al. 2013; Saito et al. 2013). For the 3′ end filtering (step 6 above), we kept all reads that overlapped with a stop codon in the WormBase WS265 GFF3 annotation (Lee et al. 2018; WormBase web site [https://wormbase.org], release WS265 2018). For those reads that did not overlap with an annotated stop codon, we examined the read for canonical or alternative polyadenylation signals (PAS) up to 60 bp upstream of the putative 3′ end, as well as a predicted open reading frame (ORF) in the read with defined start and stop codons. We kept all reads that had both an ORF and a canonical or alternative PAS.

Our collection of filtering steps ensures that we keep only full-length transcripts with 5′ and 3′ ends that correspond to TSSs and polyadenylation sites, respectively, for further analysis. To determine the efficacy of this filtering approach, we made an aggregate plot of normalized coverage across the average coding gene (Fig. 1B). Supporting the validity of this approach, the reads that fail our filtering steps have an extreme 3′ bias, while those that pass the filtering steps do not have this 3′ bias in the total reads. Passing reads comprise the majority of reads in each data set (Fig. 1C). Combining all data sets, almost 2.9 million passing reads were obtained (Supplemental Table 2 for a breakdown of reads remaining after each filtering step). Following read filtering, reads were assigned to the splice isoforms and 3′ UTRs present in each developmental stage and across all stages, as described in Methods.

### Examining read and isoform length distributions

Part of the appeal of long-read RNA sequencing is the ability to capture full-length isoforms. However, as our library preparation is dependent on the poly(A) tail, 3′-biases may skew the resulting isoform length distribution and annotation of the transcriptome. To characterize this in our data sets, we plotted the length distribution of poly(A)-selected RNA from each of our stages as identified through TapeStation traces (Supplemental Fig. 2A). We then compared the TapeStation traces to the expected fluorescence signal based on the read lengths obtained from our nanopore sequencing experiments (determining expected fluorescence by weighting by the length of the reads) (Supplemental Fig. 2B). The expected fluorescence based on the sequencing read length distribution obtained is shorter than the distribution one would expect from an unbiased sequencing experiment based on TapeStation traces. However, we identified the two major peaks in our RNA length distributions as those corresponding to ribosomal subunits, indicating that oligo d(T) pull down of RNA failed to remove all ribosomal RNA from our samples. We next sought to determine if this read length bias was resulting in a shorter identified transcriptome on average compared to the existing transcriptome annotation (Fig. 1E; Supplemental Fig. 3A), and to transcriptome annotations assembled by StringTie2 (Kovaka et al. 2019) using (1) previously collected Illumina RNA-seq data from across *C. elegans* development generated by the modENCODE Project (Hillier et al. 2009), (2) previous work from our laboratory (Weiser et al. 2017), and (3) the Albritton et al. (2014) study (Fig. 1F; Supplemental Fig. 3B). We find that despite the length difference observed between the TapeStation and nanopore read length, the length distribution of the unique isoforms we identify in our analyses are similar to the length distributions of the WormBase transcriptome annotation and transcriptome annotations produced by Illumina data and StringTie2. Taken together, these analyses indicate that our analysis pipeline mitigates the impact of any fragmentation-induced read length biases present in our sequencing and suggests that the full-length transcript isoforms we identify accurately reflect the structure and length of transcripts in the full-length *C. elegans* transcriptome.

### Identifying the full-length transcriptome

The full-length single-molecule resolution of nanopore sequencing means that, unlike short-read sequencing, the full linear sequence of exons comprising a transcript and all of the associated splice junctions (i.e., the splice isoform) and the 3′-UTR isoform are captured together in a single read. This enables the identification of the “full-length transcriptome,” the set of full-length isoforms (splice isoform + 3′-UTR isoform) observed together across all reads (Fig. 1G). When considered across all developmental stages and conditions, 28,858 full-length isoforms were identified, comprised of 23,865 unique splice isoforms and 16,342 unique 3′ UTRs (Fig. 1H; Supplemental Table 3 for exact values). Over 12,000 full-length isoforms were identified in each stage. Because 3′ UTRs were only called if there were three or more reads supporting the putative cleavage site, not all splice isoforms have an associated 3′ UTR called. Therefore, some full-length isoforms have no high-confidence 3′-UTR call and are, in effect, simply splice isoforms. This describes only a fraction (5583, 19%) of the full-length isoforms identified.

To determine if these data sets were at or approaching saturation in the number of full-length isoforms identified, reads were randomly subsampled and the number of full-length isoforms that had support from one or more reads in the subsampled set was determined. These values were then plotted, and the relationship between the number of reads considered and the number of full-length isoforms supported was examined. As expected, none of the developmentally staged data sets appears to be saturated (Supplemental Fig. 4A). We also examined the number of isoforms identified across all stages, which also does not yet appear to be saturated (Supplemental Fig. 4B).

The ability to resolve splice isoforms and 3′-UTR isoforms together at single-molecule resolution allows for identification of genes where the two features appear to be correlated. Few examples of significant correlations between splice isoform use and 3′-UTR isoform use were identified by Fisher's exact test after multiple hypothesis testing correction (Supplemental Table 4). This is possibly due to lack of coverage but more likely reflects an overall lack of coordination between splicing and polyadenylation site choice in *C. elegans*.

### Quantifying genes and splice isoforms captured with full-length support

**Figure 2.**
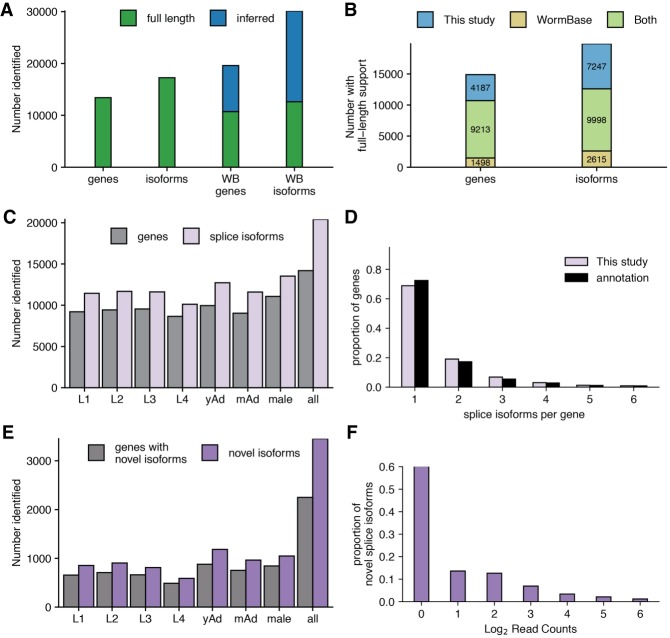
Capture of annotated and novel full-length splice isoforms. (*A*) Number of gene and isoform captures with full-length support in our data set (*left*) versus the WormBase (WB) annotation (*right*) (Lee et al. 2018; WormBase web site [https://wormbase.org], release WS265 2018). (*B*) Stacked bar graph showing overlap between isoforms and genes with full-length support in our data set and those with full-length support in the WormBase annotation. (*C*) Number of previously annotated splice isoforms and corresponding genes identified by our data across all stages. (*D*) Density plot showing the number of isoforms identified per gene across our full data set and the WormBase annotation. (*E*) Number of novel isoforms and genes with novel isoforms identified across all stages. (*F*) Density plot showing the proportion of novel splice isoforms with a given number of reads supporting their structure.

Less than half of the 30,133 isoforms with annotated introns in the WormBase WS265 annotation have full-length support (here, full-length support means that every annotated intron in the isoform is supported by the same cDNA or EST) (Fig. 2A; Lee et al. 2018; WormBase web site [https://wormbase.org], release WS265 2018). By comparison, 17,245 splice isoforms (of the 30,133 with annotated introns in WormBase) across 13,400 genes had full-length support using our data, well above the 12,613 isoforms and 10,711 genes that have full-length support in the WormBase WS265 annotation (Fig. 2A). Comparing the genes and isoforms with full-length support in each data set, 4187 genes and 7247 isoforms were identified that did not previously have full-length support (Fig. 2B). This data set therefore significantly expands the number of *C. elegans* genes and isoforms supported by full-length data.

To examine the changes of splice isoform usage in each developmental stage and across all stages, we plotted the number of previously annotated splice isoforms and genes observed in each stage (Fig. 2C; Supplemental Table 3). We found more than 10,000 previously annotated splice isoforms in each stage, with males having the most identified genes and splice isoforms of any individual stage despite having fewer reads after our filtering steps than most other stages (Supplemental Table 2). Combining across all stages, 20,413 previously annotated splice isoforms were observed. Most genes in our transcriptome data have only a single identified splice isoform, and the frequency of genes with a given number of isoforms decreases as the number of isoforms increases, consistent with the WS265 annotation of the *C elegans* transcriptome (Fig. 2D).

In addition to capturing previously annotated splice isoforms, part of the appeal of long-read single-molecule sequencing is the ability to detect novel splice isoforms. To test our ability to identify novel splice isoforms after stringent filtering and splice site correction steps, we searched for isoforms with a set of splice junctions not present in the WormBase WS265 annotation. Across all stages, 3452 novel splice isoforms were identified corresponding to 2251 genes (Fig. 2E; Supplemental Table 3). Of the novel splice isoforms, 1285 have novel splice junctions between previously annotated donor and acceptor splice sites, and 262 have novel exons. To determine the level of support for these novel isoforms, we generated a density plot showing the proportion of novel isoforms with a given number of reads supporting them (Fig. 2F). The majority of identified novel splice isoforms were identified with a single read supporting their structure; however, over 20% of novel isoforms had four or more reads supporting them, indicating that these are high-confidence novel isoforms.

Finally, we sought to examine how many of our identified splice isoforms were predicted to be noncoding using the protein coding prediction algorithm CPAT trained on the *C. elegans* transcriptome annotation (Wang et al. 2013). Using this software, 1623 of our 23,865 splice isoforms appear to be noncoding (using a threshold of coding probability of 0.5 for defining the boundary between coding and noncoding isoforms, the IDs of which are listed in Supplemental Table 5).

### Characterizing the identified 3′ UTRome

**Figure 3.**
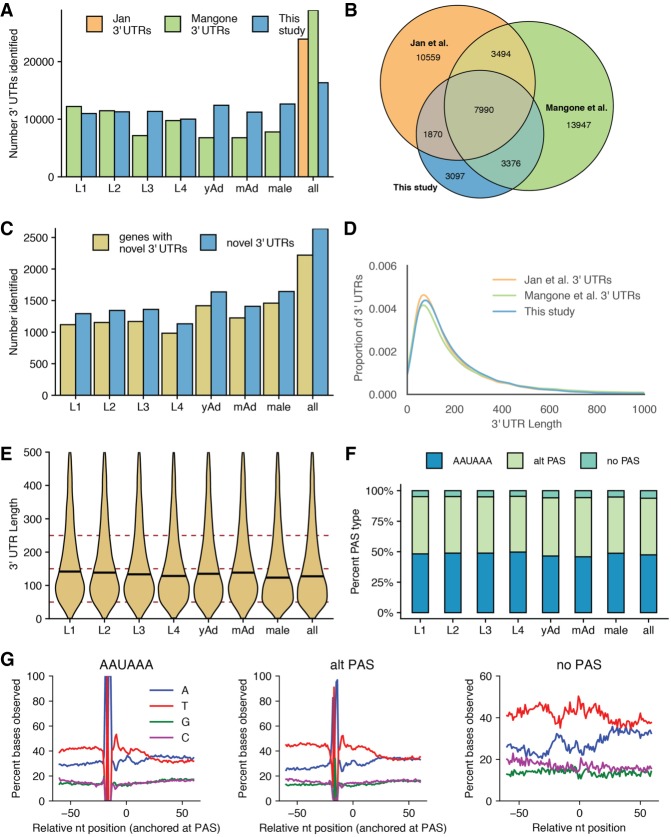
Properties of 3′ UTRome (*A*) Number of 3′ UTRs observed across all stages, as compared to Mangone et al. (2010) and Jan et al. (2011). (*B*) Venn diagram showing overlap between 3′ UTRs identified in this study, Jan et al., and Mangone et al. (*C*) Number of novel 3′ UTRs and genes with novel 3′ UTRs identified in each stage and across all stages. (*D*) Kernel density estimate plot of 3′-UTR lengths from this study, Jan et al., and Mangone et al. (*E*) Violin plots showing 3′-UTR length distributions across all stages. Horizontal black lines show the median of each stage. (*F*) Stacked bar chart showing percentage of UTRs with the specified polyadenylation signal (PAS) across all stages. (*G*) Nucleotide distributions around putative PAS sites and putative cleavage sites. Canonical PAS (AAUAAA) and alternative PAS (alt PAS) distributions are anchored with the putative PAS hexamer at −19 nt. The distribution of UTRs with no PAS is anchored with the putative cleavage site at 0.

Previous analyses of nanopore sequencing have largely centered on splice isoform identification and characterization while largely ignoring the 3′ UTR. Because dRNA-seq relies on sequencing in the 3′ to 5′ direction of mRNAs isolated by their poly(A) tails, full-length sequences of 3′ UTRs are preferentially captured. After adapter trimming, discarding reads with large 3′ softclips, and realigning the 3′ softclipped portions of the remaining reads, we identified putative poly(A) cleavage sites and predicted stop codons to define full-length 3′ UTRs. Using this method, 16,342 unique 3′-UTR isoforms were identified, with over 10,000 3′ UTRs identified in each stage (Supplemental Table 3). When splice structure in the 3′-UTR region is ignored to ease comparison with existing data sets (as described in Methods), 16,333 3′ UTRs are identified (Fig. 3A).

To determine the accuracy of our 3′-UTR calling, we compared the 3′ UTRs identified by this method with those from previously published data sets (including 3P-Seq and 3′RACE data) generated in *C. elegans* (Mangone et al. 2010; Jan et al. 2011). Of our identified 3′ UTRs, 81.0% overlap with one or more of these 3′-UTR data sets (Fig. 3B). In addition, we identified 2640 novel 3′ UTRs that do not fall within 10 bp of existing 3′ UTRs or WormBase 3′-UTR annotations (Fig. 3C). The 3′-UTR length distribution in our data was nearly identical to those observed by Jan et al. and Mangone et al. (Fig. 3D). In agreement with Mangone et al., our 3′-UTR length distributions change over developmental stages, progressively decreasing from L1 through L4, and are shorter in males than in hermaphroditic adults (Fig. 3E). The 3′-UTR length distributions in adult stages were slightly longer than the length distribution of L4 3′ UTRs in our data sets, in contrast to Mangone et al. (2010), which showed that adult 3′ UTRs had a slightly shorter average 3′-UTR length than L4.

Given that the lengths of 3′ UTRs change during development, we investigated whether PAS usage might also vary across time. We compared the frequency of canonical PAS usage (defined by the motif AAUAAA), alternative PAS usage (defined by a subset of hexamers with a 1- or 2-nt difference from AAUAAA [see Methods]), and sites with no defined PAS. Frequency of canonical and alternative PAS usage was quite consistent between adjacent developmental stages, although by χ^2^ tests, there were statistically significant differences in overall PAS usage between the L4 and young adult stage as well as between hermaphroditic young adults and males (Fig. 3F). Given that distribution of canonical and alternative PAS usage is consistent across the larval stages, where a significant shift in 3′-UTR length distributions occurs, this suggests that 3′-UTR length changes over development are largely independent of PAS usage.

As a final metric for the accuracy of this 3′ UTRome, we plotted nucleotide distributions in windows around identified PAS sites and around putative cleavage sites (Fig. 3G). This largely agrees with previously published nucleotide distributions in windows around identified PAS sites (Mangone et al. 2010). These distributions are AT-rich, with a peak in T frequencies just 3′ from the PAS site. It is possible that 3′ UTRs identified by our method were inaccurate and broadly distributed around true cleavage sites, and by anchoring nucleotide distributions with putative PAS sites at −19 nt, the impact of these errors was eliminated. To test this possibility, we generated a density plot of the offsets of identified PAS sites from putative cleavage sites identified by our method and found that these offsets were enriched close to the canonical −19 nt from putative cleavage sites, indicating cleavage site calls from this method are accurate within a few base pairs (Supplemental Fig. 5A,B).

At 3′-UTR sites without a putative PAS identified, the nucleotide distribution observed lacks the enrichment of As in a window around the cleavage site noted in Mangone et al. (2010). Our method may be capturing a different set of 3′ UTRs with no PAS than the Mangone et al. data set. Supporting this possibility, only 28% of the no-PAS 3′ UTRs in our data set overlap with a Mangone et al. 3′ UTR, as compared with 71% of canonical PAS and 64% of alternative PAS 3′ UTRs in our data (Supplemental Fig. 5C). In addition, no-PAS 3′ UTRs that do overlap with a Mangone et al. 3′ UTR have a different nucleotide distribution than the no-PAS Mangone et al. 3′ UTRs in general (Supplemental Fig. 5D; Mangone et al. 2010).

### Properties of poly(A) tail lengths

Poly(A) tails are known regulators of translation and transcript stability. However, profiling of poly(A) tail lengths at the transcriptome-wide level using short-read sequencing is a relatively recent advance in the field (Chang et al. 2014; Subtelny et al. 2014; Lim et al. 2016). We have previously shown that, using a trained hidden Markov model, one can estimate the poly(A) tail length of dRNA-seq reads using nanopolish (Workman et al. 2019). We performed these estimations on our current data, providing a developmentally resolved poly(A) profiling data set.

**Figure 4.**
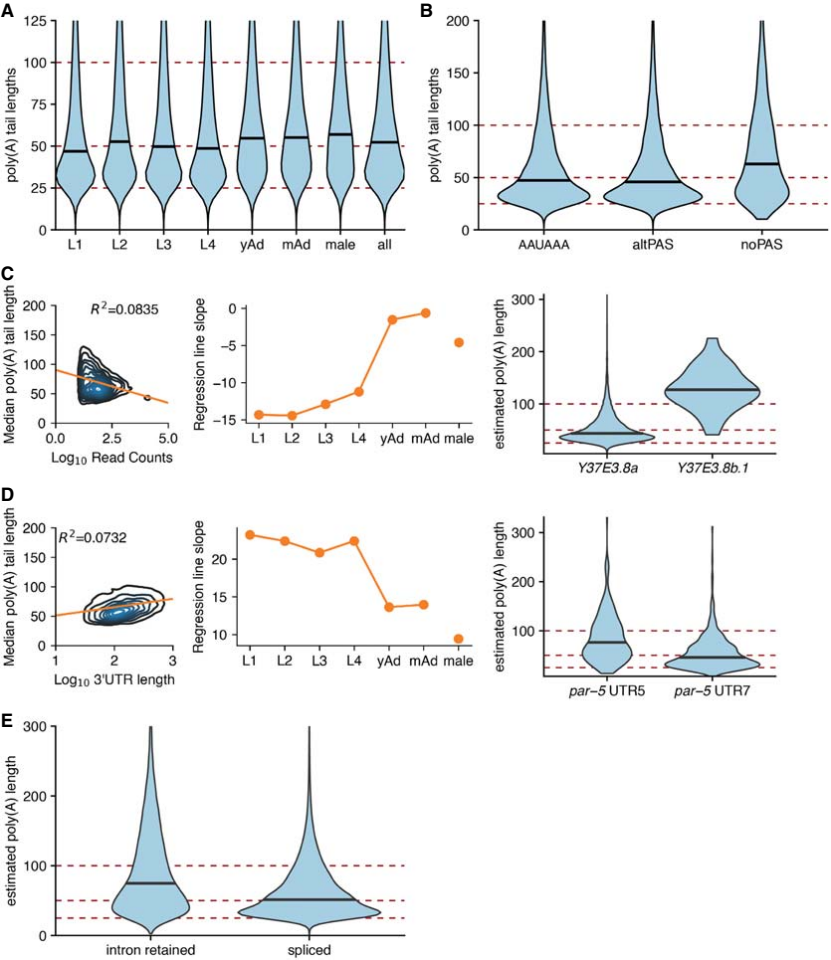
Properties of poly(A) tail length. (*A*) Violin plot of poly(A) tail length distributions across development. Horizontal black lines show the median of each stage. (*B*) Poly(A) tail length distributions separated by the PAS type of the associated reads for reads corresponding to isoforms predicted to be coding. (*C, left*) Density plot showing correlation between poly(A) tail length and expression level by plotting median poly(A) tail length for each isoform versus the log of the expression level of that isoform (across all stages). Linear regression plotted in orange. (*Middle*) Slope of linear regressions performed on median poly(A) tail length versus expression level data across developmental stages. (*Right*) Example locus illustrating relationship between poly(A) tail length and expression level *Y37E3.8b.1* is less expressed than *Y37E3.8a* with a longer poly(A) tail length distribution. (*D, left*, *middle*) As in the *left* and *middle* panels of *C*, but instead plotting median poly(A) tail length versus the log of the 3′-UTR length. (*Right*) Example locus illustrating the relationship between 3′-UTR length and poly(A) tail length; *par-5* UTR 5 is longer than *par-5* UTR 7 and has a longer poly(A) tail length distribution. (*E*) Violin plots showing poly(A) tail length distributions in fully spliced versus intron retention transcripts.

Global poly(A) tail length distributions are dynamic in the developing *Drosophila melanogaster* oocyte and embryo (Lim et al. 2016). To determine if there were comparable shifts in our poly(A) tail length distributions, we examined poly(A) tail lengths across the developmental stages in *C. elegans*. The poly(A) tail length distributions display only modest fluctuations, ranging from median values of 49 nt (L1) to 54 nt (L2) during larval development, although these shifts were considered to be statistically significant by Kolmogorov–Smirnov and Mann–Whitney *U* tests (Fig. 4A). However, length distribution in all adult stages (young and mature hermaphrodites and males) are consistently longer than in the larval stages, with a median length of 58 nt in adults compared to an aggregate median length of 52 nt across all larval stages (*P* < 2.2 × 10^−16^ by Kolmogorov–Smirnov and Mann–Whitney *U* tests). These data suggest that the most significant regulation of poly(A) tail lengths occurs between larval and adult stages during development.

As a means of confirming the validity of our poly(A) tail length profiling approach, we compared our poly(A) estimates from the L4 stage with previously published poly(A) measurements from the L4 stage of *C. elegans* from mTAILseq (Lima et al. 2017). The length scale distributions of our L4 data and the Lima et al. data set are quite similar, as both have peaks around 30–40 nt and extended toward the longer tail length range (Supplemental Fig. 6A). However, we did not identify the shoulder peaks present in the Lima et al. data set (Lima et al. 2017).

An advantage of profiling poly(A) tail lengths with dRNA-seq versus short-read sequencing is that poly(A) tail lengths are directly coupled to information about the splice isoforms and 3′-UTR isoforms of the associated read. This allows comparisons and correlations between poly(A) tail lengths and aspects of transcript structure. One possible driver of differences in poly(A) tail lengths between reads could be that poly(A) tail length distributions may vary depending on whether the associated 3′ UTR has a canonical PAS site. To test this possibility, we plotted poly(A) tail length distributions versus PAS type (i.e., canonical AAUAAA, alternative PAS, and no PAS) for reads from the L1 stage corresponding to isoforms predicted to be coding (based on the CPAT prediction algorithm [Fig. 4B; Wang et al. 2013]). We find that all PAS types are significantly different from one another by Kolmogorov–Smirnov and Mann–Whitney *U* tests (*P* < 2.2 × 10^−16^), and 3′ UTRs with no PAS have longer poly(A) tail lengths, on average, than poly(A) tails associated with either canonical and alternative PAS, with a median poly(A) tail length of 62 nt for 3′ UTRs with no PAS, 46 nt for 3′ UTRs with alternative PAS, and 48 nt for 3′ UTRs with canonical PAS.

It has been reported that median poly(A) tail length and expression level are anticorrelated, such that highly expressed genes generally have shorter median poly(A) tail lengths (Lima et al. 2017; Legnini et al. 2019). To determine if this relationship holds in our data sets, we plotted the log of the number of reads supporting a given isoform versus the median poly(A) tail length for that isoform for transcripts with 10 or more reads supporting them (Fig. 4C, left panel; Supplemental Fig. 6B). A similar inverse correlation between median poly(A) tail length and number of reads supporting that isoform was observed in the L1 to L4 stages and when all stages were pooled (Supplemental Fig. 6B). For example, the *a* isoform of the *Y37E3.8* gene (*Y37E3.8a*) is expressed much more than the *b.1* isoform (*Y37E3.8b.1*; 13,299 reads vs. 26 reads) and has a significantly shorter poly(A) tail length distribution than the *b.1* isoform (Fig. 4C, right panel). However, this correlation explains only a small fraction of the overall variation in the data, with the maximum *R*^2^ value of 0.1242. In the adult stages (both males and hermaphrodites), the slope of the regression lines between median poly(A) tail length and expression level were much shallower, and the corresponding *R*^2^ values were much weaker, with *R*^2^ values ranging from 0.0003 to 0.0122 (Fig. 4C, middle panel; Supplemental Fig. 6B). These results suggest that the inverse relationship between poly(A) length and expression level may vary depending on the developmental stage.

A recent study using FLAM-seq, a Pacific Biosciences (PacBio) sequencing method that also captures poly(A) tails and full-length transcripts, demonstrated that poly(A) tail length and 3′-UTR length were positively correlated (Legnini et al. 2019). Examining poly(A) tail length and 3′-UTR lengths across all reads in our data, we also identify this same relationship (Fig. 4D, left panel). For example, the longer *par-5* 3′-UTR isoform (termed 3′ UTR 5; 486 nt) also has a longer poly(A) tail (median length 74 nt) versus the shorter *par-5* 3′-UTR isoform (3′ UTR 7; 51 nt) with a shorter poly(A) tail length distribution (median length 45 nt) (Fig. 4D, right panel). However, the overall strength of this relationship also varies between developmental stages, and the slopes of the regression lines (and the corresponding *R*^2^ values) are smaller in adult stages than in larval stages (Fig. 4D, middle panel; Supplemental Fig. 6C).Finally, we examined the poly(A) tail length distributions between transcripts that are fully spliced versus those with retained introns (Fig. 4E). We previously showed in the human cell line GM12878 that intron retention correlates with transcripts with longer poly(A) tails (Workman et al. 2019). In our *C. elegans* data sets, we also found a positive correlation between intron retention and poly(A) tail length distributions by Kolmogorov–Smirnov and Mann–Whitney *U* tests, suggesting a conserved mechanism whereby nuclear transcripts possess longer poly(A) tails and supporting a model proposed by Lima et al. (2017) in which poly(A) tails may be subject to post-transcriptional processing by deadenylation once exported into the cytoplasm.

### A public resource for full-length isoform information

**Figure 5.**
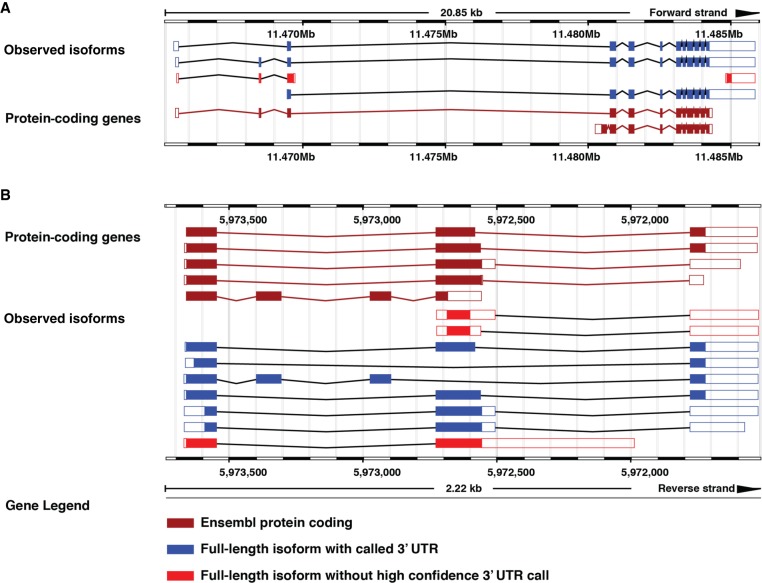
Examples highlighting the utility of our custom track hub. The *lin-14* (*A*), or *mlp-1* (*B*) locus in the Ensembl Genome Browser including our custom track hub. Blue isoforms are full-length isoforms with an associated 3′ UTR called; red isoforms have no high-confidence 3′ UTR called. Burgundy isoforms are protein-coding models imported from WormBase.

To make our transcriptome data set accessible to the research community, we have created a public custom track hub (https://bx.bio.jhu.edu/track-hubs/dRNAseq/hub.txt). This track hub contains the full-length filtered and nonfiltered reads from each developmental stage, as well as the full-length isoforms supported across all stages at each locus. To ease access to this track hub, we registered it with the Track Hub Registry (https://trackhubregistry.org). Users can therefore easily load this track hub in Ensembl-based genome browser (Zerbino et al. 2018) by searching public track hubs for “ce11 staged dRNAseq”. As a proof of the utility of this track hub, we loaded the track hub in the Ensembl Genome Browser and searched for *lin-14*, a gene with a well-studied 3′ UTR that is subject to regulation by the *lin-4* microRNA (Wightman et al. 1991, 1993; Lee et al. 1993) but whose 3′ UTR is not currently annotated in the WormBase WS265 annotation (Lee et al. 2018; WormBase web site [https://wormbase.org], release WS265 2018). In our data set, we identified the *lin-14* 3′ UTR, as well as its splice isoforms, including multiple novel splice isoforms (Fig. 5A, “observed isoforms” track). As another example of the utility of this track hub, we searched for the locus *mlp-1*, a gene with multiple splice and 3′-UTR isoforms identified, including multiple novel splice isoforms (isoforms 1, 2, 4, 5, and 9 of the observed isoform track in Fig. 5B). These examples highlight possible uses of this resource by the research community to query currently unannotated 3′ UTRs and splice isoforms.

## Discussion

Despite years of study, our understanding of the *C. elegans* transcriptome remains incomplete. Although studies have been performed profiling transcription start sites, splicing in both *cis* and *trans*, 3′-UTR isoforms, poly(A) tail lengths, RNA base modifications, and gene and isoform expression levels, the short read lengths intrinsic to the prevailing technologies have limited the examination to one or two of these features at a time (Hillier et al. 2009; Mangone et al. 2010; Jan et al. 2011; Saito et al. 2013; Zhao et al. 2015; Lima et al. 2017; Tourasse et al. 2017; West et al. 2018; Packer et al. 2019). Even within these data sets, short read lengths and reliance on PCR amplification eliminate single-molecule resolution and make correlation of distant features within transcripts impossible. Although our study focuses primarily on splice isoforms, 3′-UTR isoforms, and poly(A) tail lengths due to current limitations of nanopore sequencing technologies, in principle, modified approaches to dRNA-seq would be capable of capturing all of the above features at a single-molecule level.

Nanopore sequencing therefore poses both a unique set of opportunities and challenges that must be addressed in any analysis pipeline. The dRNA-seq pipeline FLAIR (full-length alternative isoform analysis of RNA) utilizes a hybrid sequencing approach in which matched short-read sequencing is used to correct splice junctions in reads, and reads are clustered together into splice isoforms if they share a common set of splice junctions (Tang et al. 2018).

We utilized an approach similar to that used by FLAIR, in which reads are corrected, in our case by an existing annotation, and clustered together by splice isoform. Our approach differs from FLAIR in several ways, including a series of filtering steps that reduces the impact of 3′ bias in our reads and allows us to consider only full-length transcripts. A recent publication examining the utility of dRNA-seq and cDNA nanopore sequencing to generate transcriptome annotations independently revealed that many nanopore sequencing reads fail to span the full-length of annotated transcript isoforms, highlighting the need for analysis pipelines that take the possibility of 5′ truncations into account in isoform identification (Soneson et al. 2019). Our full-length filtering approach partially addresses this concern, although, as noted by Soneson and colleagues, doing so reduces the number of usable reads and likely impacts the quantitative nature of our data. A possible experimental approach to solving this problem could involve ligating a set of known nucleotides to the 5′ end of RNA transcripts after a decapping reaction, allowing for selection of full-length transcripts by filtering for reads flanked by signals corresponding to a poly(A) tail and the 5′ ligated product. This approach would incidentally also address the known problem of the 10–15 nt at the 5′ end of each strand that are unable to be read by nanopore sequencing methods (Workman et al. 2019).

Also distinguishing our approach from FLAIR is a novel means of calling 3′ UTRs used in the generation of transcriptome annotations. We identify 3′-UTR structures with a standard dRNA-seq library preparation protocol, meaning that, in principle, any dRNA-seq experiment can be used to identify 3′ UTRs using our method. The implications of this are potentially wide-reaching, as experiments once used for comparative analysis of splice isoforms between conditions may now also be used in comparative analysis of 3′-UTR isoforms.

By combining our 3′-UTR and splice isoform calls, we identified over 28,000 full-length transcript isoforms. It is likely that increased depth and additional sequencing of other developmental stages such as embryos and the stress-induced dauer stage would further increase the number of genes and isoforms identified, bringing this data set closer to capturing the theoretical complete *C. elegans* transcriptome.

The ability to estimate poly(A) tail lengths for each read is another advantage of dRNA-seq. Supporting the validity of our poly(A) profiling approach, the length distribution of the poly(A) tail length estimates we obtain in the L4 stage are quite similar to the distribution in the L4 stage reported by Lima et al., a study utilizing mTAIL-seq (Lima et al. 2017). Coupling of poly(A) tail lengths to aspects of 3′-UTR structure and splice isoform allowed us to identify relationships between putative PAS sites and intron retention transcripts to poly(A) tail lengths. The relationship between PAS sites and poly(A) tail lengths is a result that indicates there may be differential deposition or regulation of poly(A) tail length based on the presence or absence of an upstream PAS sequence. Longer poly(A) tails in intron retention transcripts could be indicative of partially processed RNAs retained in the nucleus, as nuclear RNAs would be shielded from cytoplasmic deadenylation. Neither of these relationships could have been discovered by short-read sequencing of poly(A) tails, demonstrating the efficacy of full-length single-molecule sequencing.

One discovery of developmentally resolved poly(A) tail length profiling was the difference in features of poly(A) tail lengths between larval and adult stages. Overall, poly(A) tail length distributions were longer in adult stages than in larval stages, and the strength of previously reported correlations between poly(A) tail lengths and expression level and poly(A) tail lengths and 3′-UTR lengths were weaker in adult stages than larval stages. One possible explanation for these differences is the development of a functional germline in adult stages. In hermaphrodites, the cytoplasmic polyadenylases *gld-2* and *gld-4* are known to be active in the germline (Suh et al. 2006; Schmid et al. 2009; Millonigg et al. 2014; Nousch et al. 2017). Given the relative size of the *C. elegans* germline, it is possible that activity of such cytoplasmic poly(A) polymerases may influence global poly(A) tail length distributions.

Finally, we have created a custom track hub for exploration of this data set by independent researchers. By making this data easily accessible, we hope to provide *C. elegans* researchers with information related to their genes of interest, providing a resource to identify what isoforms have full-length support in any given developmental stage, and across all stages, as well as the structure of any 3′ UTRs that we identify. Given that our data set provides support for over 7000 isoforms previously lacking full-length support and over 23,000 splice isoforms overall and given that most isoforms have an associated 3′ UTR called, this will be a resource for the *C. elegans* research community. Overall, we have demonstrated the utility of nanopore sequencing in providing support for full-length transcripts, annotating putative 3′ UTRs, and interrogating poly(A) tail lengths.

## Methods

### *C. elegans* strains, maintenance, and collection

*C. elegans* N2 worms were grown and maintained under standard laboratory conditions on NGM plates seeded with *Escherichia coli* OP50 (Stiernagle 2006). Samples for RNA analysis were synchronized by hypochlorite treatment and overnight hatching in M9 buffer. They were plated as starved L1 diapause worms at 25°C and staged by pharyngeal pumping. L2, L3, L4, and young adult (YA) worms were collected ∼2 h post-lethargus. L1 worms were collected 4 h after plating. Mature adults were collected ∼10 h post-L4/YA transition. CB1489 [*him-8(e1489)*IV] adult males were enriched by filtering through 35-µm mesh.

### RNA extraction

Total RNA isolation was performed using TRI Reagent (Ambion) following the vendor's protocol, with the following alterations: Three rounds of freeze/thaw lysis were conducted prior to the addition of BCP; RNA was precipitated in isopropanol supplemented with glycogen for 1 h at −80°C; RNA was pelleted by centrifugation at 4°C for 30 min at 20,000*g*; the pellet was washed three times in 70% ethanol; the pellet was resuspended in water.

### Library preparation and sequencing

Approximately 20-μg aliquots of total RNA were diluted to a total volume of 100 μL in nuclease-free water and poly(A)-selected using NEXTflex Poly(A) Beads (BIOO Scientific, Cat#NOVA-512980). Up to 600 ng of the resulting poly(A) RNA was separately aliquoted for library generation. Any excess poly(A)-selected RNA was stored at −80°C. Biological poly(A) RNA and a synthetic control (Lexogen SIRV Set 3, 2.5 ng) were prepared for nanopore direct RNA sequencing generally following the Oxford Nanopore Technologies (ONT) SQK-RNA001 kit protocol, including the optional reverse transcription step recommended by ONT. One difference from the standard ONT protocol was use of SuperScript IV (Thermo Fisher Scientific) for reverse transcription. RNA sequencing on the GridION platform was performed using ONT R9.4 flow cells and the standard MinKNOW protocol script (NC_48Hr_sequencing_FLO-MIN106_SQK-RNA001).

### Preprocessing and alignments

Reads were base-called and trimmed of adapter sequences using Poreplex version 0.3.1 (running Albacore version 2.3.1) with the following parameters: -p 24 --trim-adapter --basecall (https://github.com/hyeshik/poreplex). For each of our samples, reads were aligned to the WBcel235 ce11 genome using minimap2 version 2.14-r883 (Li 2018). Genomic alignments were run with the following parameters: -ax splice -k14 -uf --secondary = no -G 25000 -t 24. The resulting SAM files were converted to BAM format using SAMtools view with parameters: -b -F 2048 (Li et al. 2009).

### Read filtering

Our first filtering step involved removing reads aligning to the genome with large insertions (>20 bp) and large 3′ softclips (>20 bp) that could be the result of not properly aligning internal or 3′ exons, respectively. This filtering step ensures that novel isoforms identified in downstream scripts are not false positives resulting from poor alignments.

Following this, reads were filtered based on their QC tags from the poly(A) estimation module of the program nanopolish (Workman et al. 2019). Reads were removed from consideration if they had QC tags “READ_FAILED_LOAD”, “SUFFCLIP”, or “NOREGION”. This was meant to remove reads without a detectable poly(A) tail signal, to prevent inclusion of reads with truncated 3′ ends.

Next, for the purposes of better identifying 3′-UTR isoforms in downstream analysis, 3′ soft-clips were realigned using a semiglobal aligner with affine gap penalties anchored at the 3′ end of the original alignment. This resulted in more uniform 3′ ends of alignment. The resulting realigned reads were converted to BED12 format using the BEDTools bamtobed function (version 2.27.1) (Quinlan and Hall 2010; Quinlan 2014).

Reads were then filtered to ensure that their 5′ ends reflected a bona fide TSS. This filter selected for reads with 5′ ends either within −100 to +15 bp of an annotated 5′ end of a transcript in the WormBase WS265 GFF3 file, within 5′ SAGE, or RNA polymerase II initiation clusters from Saito et al. and Chen et al., or within 10 bp of a called TSS from the same data (Chen et al. 2013; Saito et al. 2013). Note that many of the 5′ ends of transcripts from the WS265 annotation do not reflect the true TSS of the gene but the end of outrons that are spliced out of the mature transcript at sites of *trans* splicing. Our approach, therefore, makes extensive use of *trans* splicing acceptor sites when defining full-length transcripts.

To account for errors in splice junction alignments, we used the WormBase WS265 GFF3 annotation to define canonical donor and acceptor splice sites and assigned each donor and acceptor splice site in our reads to a canonical splice site. Noncanonical donor and acceptor splice sites in our reads that fell within 15 bp of a canonical site were assigned to that site. Reads that contained noncanonical donor and acceptor splice sites that were not within 15 bp of a canonical site were discarded and not considered for the purposes of defining splice isoforms or UTRs. In addition, reads were thrown out if splice junctions in that read corresponded to annotated splice junctions from more than one gene. This allowed us to assign each spliced read to a gene based on its correspondence to annotated donor and acceptor splice sites. Reads were assigned to splice isoforms in a similar manner (however, some of these assignments were ambiguous when two annotated isoforms were comprised of the same sets of splice junctions). For nonspliced reads, we assigned gene IDs based on overlap with single exon genes present in the annotation.

Next, we separated reads that had exons that span the full length of any intron in the annotation that is not fully spanned by an exon in the annotation. We do this to separately consider intron retention transcripts when defining putative isoforms, as we believe these reads to be nuclear RNA that has not been fully processed, which, if included, would artificially inflate the number of identified isoforms. Intron retention reads are only considered in analysis of poly(A) tail length distributions, in the comparison of poly(A) tail length distributions in fully spliced versus intron retention transcripts, and as a separately reported track in our custom Track Hub.

Finally, we implemented a filter to ensure we could be highly confident in our 3′ ends. This filter first keeps all reads that overlap with an annotated stop codon (extracted from the WormBase WS265 GFF3 annotation file into a stop_codons.bed file) (as determined using BEDTools intersect with the flags -u -s -split) (Lee et al. 2018; WormBase web site [https://wormbase.org], release WS265 2018). Of the reads that do not overlap with an annotated stop codon, we search the 3′ end of the read for a putative canonical or alternative PAS, and we perform open reading frame predictions to determine if the read has a predicted open reading frame with a defined start and stop codon. If the read has both a putative PAS and a putative ORF, the read is kept; otherwise, the read is not considered in downstream analyses.

Reads were excluded from consideration in 3′-UTR calling (but not splice isoform calling) if their original minimap2 alignments had 3′ softclips larger than 10-nt long. This exclusion prevented reads with 3′ ends that failed to align well from being considered and reduced the variation in considered 3′ alignment ends significantly.

### Splice isoform identification

After the intron retention filter and before our 3′ filter, we extracted the sequences from the ce11 WBcel235 genome corresponding to each aligned read using the getfasta function of the program BEDTools with the following flags: -s -split -bedOut (Quinlan 2014). After completing the 3′ filtering step, we then clustered reads (and their associated sequences) together into putative isoforms if the reads shared a common set of splice junctions. This resulted in reads clustered by splice isoform. For each of these sets of reads corresponding to splice isoforms, we selected the longest read. From this read, we extracted information about the isoform including putative coding sequence by identifying the longest open reading frame (with both start and stop codons) present in the read's associated sequence. This allowed us to define putative start and stop codons.

Splice isoforms were called as novel if they contained a set of splice junctions not previously annotated in the reference. To deal with the possibility of 5′ truncated reads artificially inflating our novel isoform counts, we considered all possible 5′ truncations of previously annotated transcripts in the WormBase WS265 annotation file when defining our reference.

### Generating an Illumina-based transcriptome annotation with StringTie2

We utilized Illumina RNA-seq reads from across *C. elegans* development (namely L1–L4, young adult, mature adults, and males) (for accession numbers, see Supplemental Table 6). These libraries were generated by the modENCODE Project (Hillier et al. 2009), a previous publication from our laboratory (Weiser et al. 2017), and Albritton et al. (2014). Reads were aligned to the genome using HISAT2 (Kim et al. 2015) with the --dta flag. The resulting SAM alignments were converted to BAM format using SAMtools (Li et al. 2009) and provided as input into StringTie2 version 2.0 (Kovaka et al. 2019). StringTie2 was run with the WormBase WS265 GFF3 annotation file provided to guide assembly.

### 3′-UTR calling

To identify putative 3′ UTRs, reads were first grouped by their putative stop codons and any splice junctions that occurred downstream from that stop codon. For each read in each of these groups, the 3′-most base in their alignment was extracted. These end positions were then used to generate a Gaussian kernel density estimate (using the Python package Seaborn, version 0.9.0 kdeplot function with a specified kernel width of 10). Local maxima in this kernel density estimate were identified and reported as a putative 3′-UTR cleavage site if there were at least three read end positions within 10 bp of that local maxima. Reads were assigned to a given 3′ UTR if that UTR's putative cleavage site was the closest UTR cleavage site to the end position of the read and if the end position of the read and the putative cleavage site were within 10 bp of each other.

### Poly(A) tail length estimation

Poly(A) tail lengths were estimated from raw signal for each read using the poly(A) estimation function of the program nanopolish (version 0.10.2) (Workman et al. 2019). Poly(A) tail length estimates were only considered if the QC tag reported by nanopolish was PASS. Poly(A) tail length estimates were grouped by gene and isoform using the gene and isoform assignments for each read derived from comparison of genomic alignments with the splice junctions in the WormBase WS265 GFF3 reference.

### Calculating coverage for the metagene plot

To generate the metagene plot displayed in Figure 1B, we calculated coverage across every gene (as defined by the ce11 WS245 WormBase .gtf annotation file converted to BED format) using the BEDTools coverage function (Quinlan and Hall 2010; Lee et al. 2018). We then summed these coverage values together and normalized the resulting values by dividing each value by the sum of all the coverage values. Gene sizes were scaled such that the size of the gene body and the UTRs were always the same.

### Determining full-length support from WormBase annotations

A WormBase splice isoform was said to have full-length support if every one of its introns in the WS265 annotation GFF3 was annotated to have support from the same EST or the same cDNA (Lee et al. 2018; WormBase web site [https://wormbase.org], release WS265 2018). This restricted our analysis to only consider isoforms that were annotated as having introns and excluded single exon genes and genes without introns annotated in the GFF3 annotation file (which includes all noncoding RNAs). To account for this, when comparing the number of genes and isoforms we support to the number of genes and isoforms with full-length support in WormBase, we only considered splice isoforms from our data set that corresponded to an isoform from the restricted WormBase isoform set.

Annotated isoforms that lack support from full-length sequencing may still represent bona fide full-length transcripts whose annotation was derived with the aid of some degree of inference. However, without such empirical sequencing evidence, we cannot be completely confident in calling it a validated full-length transcript (see Supplemental Material for more details).

### Predicting coding potential with CPAT

We utilized CPAT (Coding-Potential Assessment Tool) to determine the number of splice isoforms we identify that are predicted to be coding, as well as to filter for reads from isoforms predicted to be coding in Figure 4B (Wang et al. 2013). To train this algorithm on the *C. elegans* transcriptome, we utilized three files from the WS265 WormBase annotation ftp site (Lee et al. 2018; WormBase web site [https://wormbase.org], release WS265 2018), the FASTA file describing CDS transcripts, the FASTA file describing mRNA transcripts, and the FASTA file describing ncRNA transcripts. We first converted all Us in the ncRNA FASTAs to Ts using sed ‘s/U/T/g’, and then used the ncRNA FASTA and the CDS FASTA in the CPAT script make_hexamer_tab.py to generate a file of hexamer counts in noncoding and coding RNA in *C elegans*. We then ran the CPAT script make_logitModel.py using the mRNA FASTA file, the ncRNA FASTA file, and the hexamer count file generated by make_hexamer_tab.py. We used the resulting model as input into cpat.py, along with the extracted sequences from each of our splice isoforms, to generate a coding potential prediction for each splice isoform we identify. We used a threshold of 0.5 as our cutoff between noncoding and coding isoforms.

### 3′-UTR comparisons

We compared our 3′ UTRs to the 3′ UTRs identified in Jan et al. (2011) and Mangone et al. (2010) using a custom script, compareUTRdatasets.py (available on the GitHub for this project https://github.com/NatPRoach/c_elegans_dRNAseq_analysis and as Supplemental Code), that required putative stop codons match identically but allowed for a 10-bp tolerance in putative 3′-UTR end positions (Mangone et al. 2010; Jan et al. 2011). Since previous studies examining 3′ UTRs would be unable to identify splicing structure within the 3′ UTR, we considered only the chromosome, start, stop, and strand of our 3′ UTRs when comparing the number and overlap of 3′ UTRs in our data set with these previous data sets. Collapsing the data in this way very slightly reduces the number of unique 3′ UTRs in our data set, hence the slight discrepancy between the number of 3′ UTRs accounted for in Figure 3, A and B, and the number of 3′ UTRs reported in Supplemental Table 3. We identified novel 3′ UTRs in a similar manner but also added consideration of WormBase annotated 3′ UTRs.

### Calling PAS sites

We identified PAS sites in a method similar to that used by Mangone et al., in which we searched the 60 nt upstream of the putative cleavage site for putative PAS hexamers (Mangone et al. 2010). Rather than recalculating the frequency of putative PAS hexamers upstream of our putative cleavage sites, we used the PAS hexamers specified in Supplemental Table 5 of Mangone et al. (2010) and searched for these hexamers in the order they appear in that table. Once a putative PAS site was identified, the UTR was assigned that PAS hexamer. If the 3′ UTR had none of the hexamers present in the table in its upstream sequence, the UTR was said to have no PAS.

### Plotting PAS nucleotide distributions

To plot the nucleotide distribution around a given type of PAS site, we first sorted sequences by their PAS type. For canonical and alternative PAS sites, nucleotide distributions were anchored such that the PAS site began at −19 nt. The percentage of use of each base at each position in a window around the PAS site was then calculated. For UTRs with no PAS identified, the nucleotide distribution was calculated such that the putative cleavage site was at position 0.

## Data access

All raw and processed sequencing data generated in this study have been submitted to the European Nucleotide Archive (ENA; https://www.ebi.ac.uk/ena) under accession number PRJEB31791. The code required to replicate the analyses performed in this paper is available as Supplemental Code, as well as on GitHub at https://github.com/NatPRoach/c_elegans_dRNAseq_analysis.

## Competing interest statement

N.P.R., N.S., and W.T. were reimbursed for conference fees, travel, and accommodation to speak at events organized by Oxford Nanopore Technologies (ONT). W.T. has two patents licensed to ONT (8,748,091 and 8,394,584).

## Supplementary Material

Supplemental Material
