# Supplementary material for: The full-length transcriptome of C. elegans using direct RNA sequencing
Source: Genome Res. 2020 Feb;30(2):299–312. doi: 10.1101/gr.251314.119 (PMC7050520; doi:10.1101/gr.251314.119)
Supplement: Supplemental Material [file supp_30_2_299__index.html]

The full-length transcriptome of C. elegans using direct RNA sequencing — Supplemental Material 

# The full-length transcriptome of *C. elegans* using direct RNA sequencing

## Supplemental Material

- Supplemental\_Code.tar.gz.zip
- Supplemental\_Material.pdf
- Supplemental\_Table\_S5.csv
- Supplemental\_Table\_S3.xlsx
- Supplemental\_Table\_S4.xlsx
- Supplemental\_Table\_S1.xlsx
- Supplemental\_Table\_S2.xlsx
- Supplemental\_Table\_S6.xlsx
