# Supplementary material for: The full-length transcriptome of C. elegans using direct RNA sequencing
Source: Genome Res. 2020 Feb;30(2):299–312. doi: 10.1101/gr.251314.119 (PMC7050520; doi:10.1101/gr.251314.119)
Supplement: Supplemental Material [file supp_gr.251314.119_Supplemental_Material.pdf]

Table of Contents:

Supplemental Discussion	Page 2
Supplemental Figure 1	Page 3
Supplemental Figure 2	Page 4
Supplemental Figure 3	Page 6
Supplemental Figure 4	Page 7
Supplemental Figure 5	Page 8
Supplemental Figure 6	Page 9

Supplemental Discussion

Regarding identification of *trans* splice sites with dRNAseq:

Splicing in *trans* is a common RNA processing event in *C. elegans*, and identifying trans splice sites would in theory be one way of identifying full-length RNA transcripts. Though it is possible to identify putative splice leader sequences at the 5′ end of transcripts with direct RNA sequencing, it is an extremely challenging and error prone task for the technology. To start, the last 10 - 15 bases at the 5′ end of each transcript are not read by the sequencer. This means, in the best case scenario, only the 12 of the 3′ most nts of the 22nt splice leader will be registered. In addition, the error rate of nanopore sequencing ranges between 10 - 15%, and as stated in the main text, in our sequencing experiments averaged at around 14%. This means that, on average, there will be at least one error in those 12 bases. These errors are predominantly insertions and deletions, error types that are essentially not considered in the logic of motif finding and motif matching approaches. All of this contributes to the difficulty of accurately determining if transcripts contain 5′ SL sequences. Thus, although one could likely identify true positive transcripts *trans* spliced to SL sequences, the lack of a matching motif does not necessarily imply the lack of *trans* splicing of that isoform, as the truncated 5′ ends and the high error rate ensures that many genuinely trans spliced transcripts will not be identified by motif searching approaches. As such, we opted not to characterize *trans* splicing in this manuscript.

Regarding the "full-length" status of transcripts in the annotation:

It should be noted that most existing annotation isoforms are likely "full-length" in that they likely represent full-length transcripts that can be expressed by the organism. However, most of these isoforms are assembled using some amount of inference because the sequencing reads used to support those isoforms are not full-length. As such, though annotation approaches have inferred that these transcripts could be expressed, for many of the annotation isoforms there is no definitive evidence that the full-length isoform is expressed. The best short-read transcriptome assemblers can do to provide support for individual isoforms longer than their read length is infer (using imperfect algorithms) which exons are spliced together in the same isoforms. This is a fundamental problem with short read transcriptome assembly and annotation that can only be addressed using long reads as done in this manuscript.

Supplemental Figures

Supplemental Figure 1

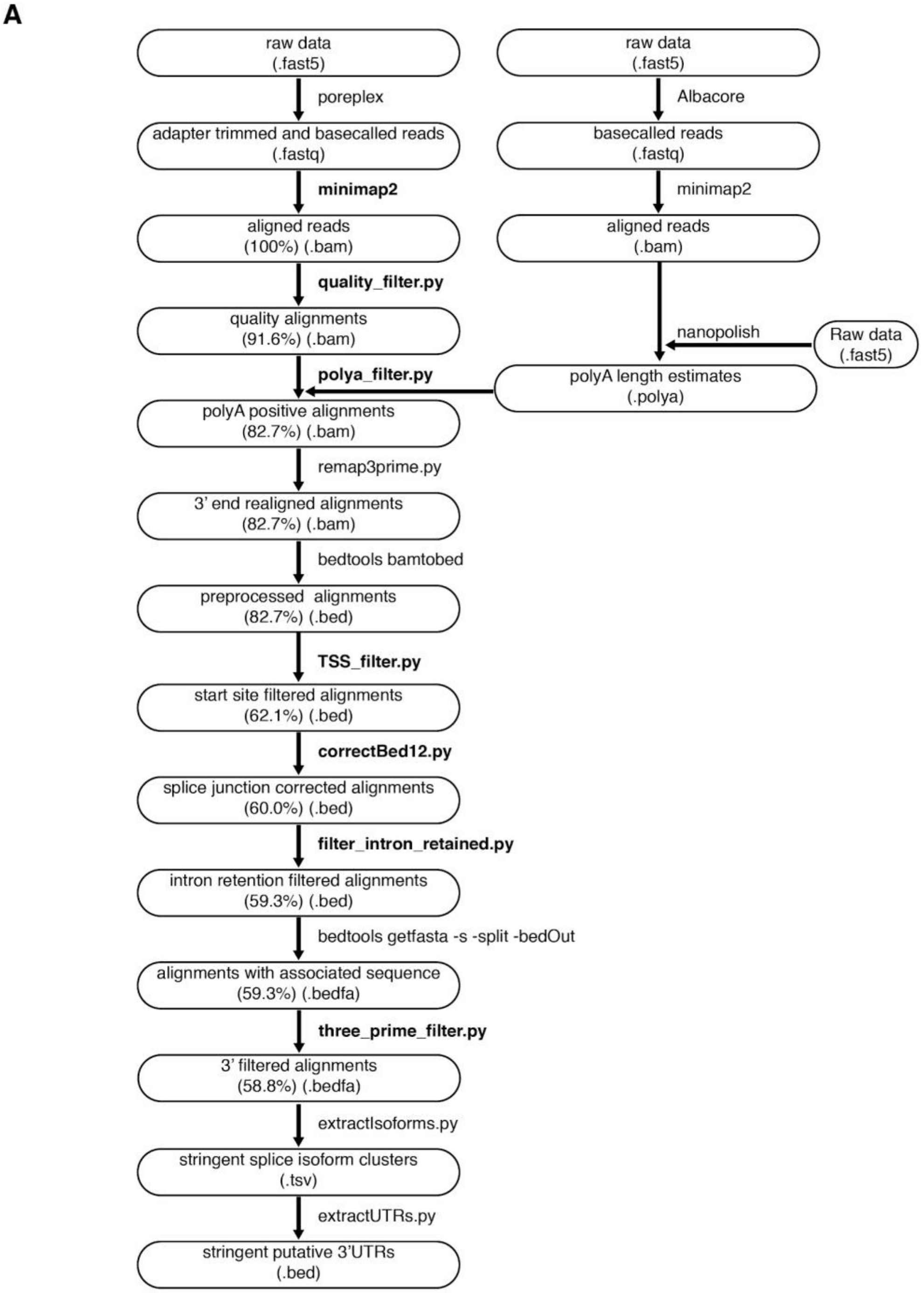

Supplemental Figure 1 - (A) Flowchart of analysis pipeline and read filtering used in this study. Percentages indicate the number of aligned reads retained up to that filtering step. File types after each step included in parenthesis.

Supplemental Figure 2

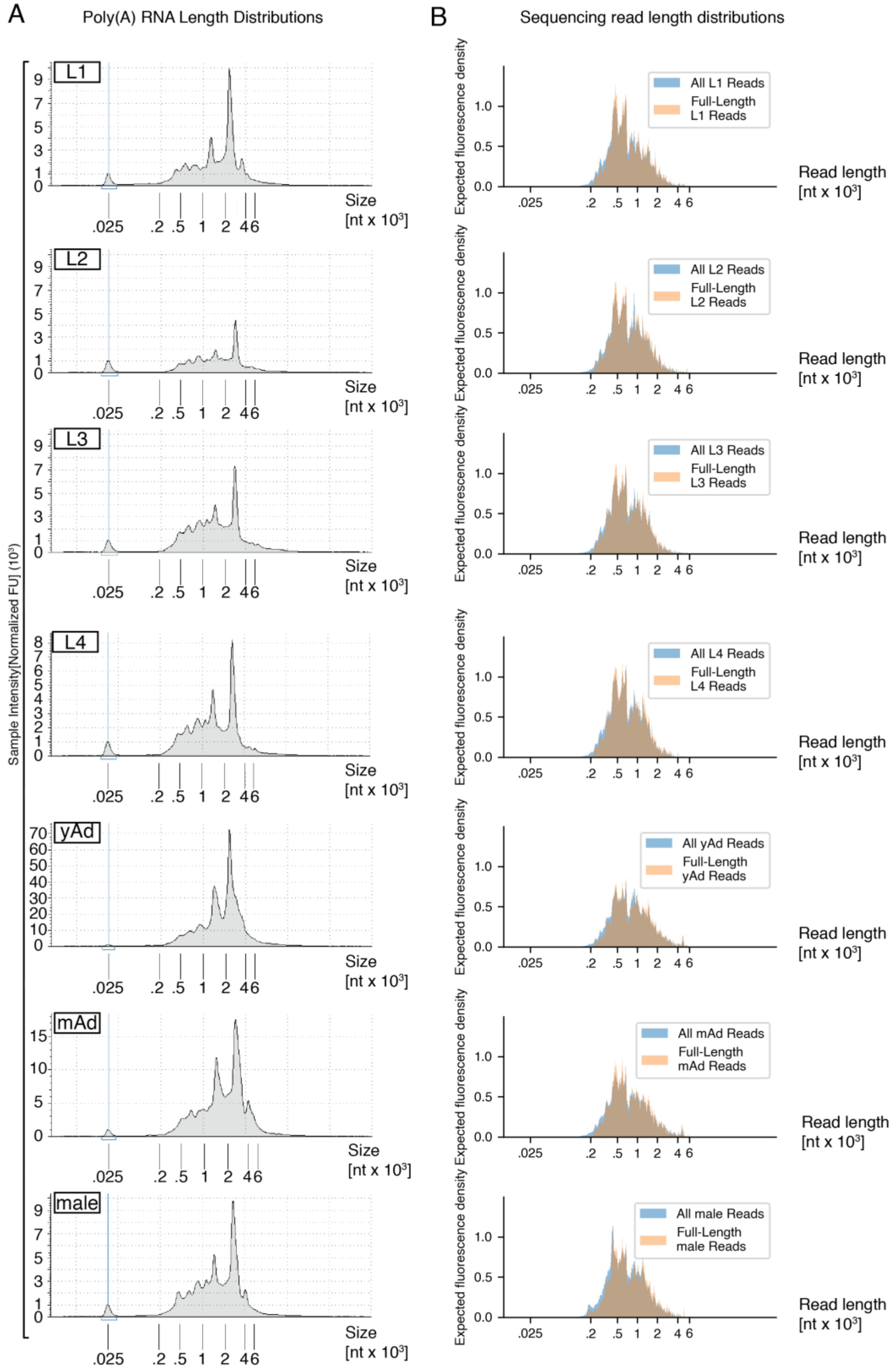

Supplemental Figure 2 - (A) TapeStation traces showing length distribution of poly(A) selected RNA from each of the developmental stages sequenced. **(B)** Expected fluorescence distribution of reads obtained from dRNAseq of each developmental stage before and after filtering steps were applied.

Supplemental Figure 3

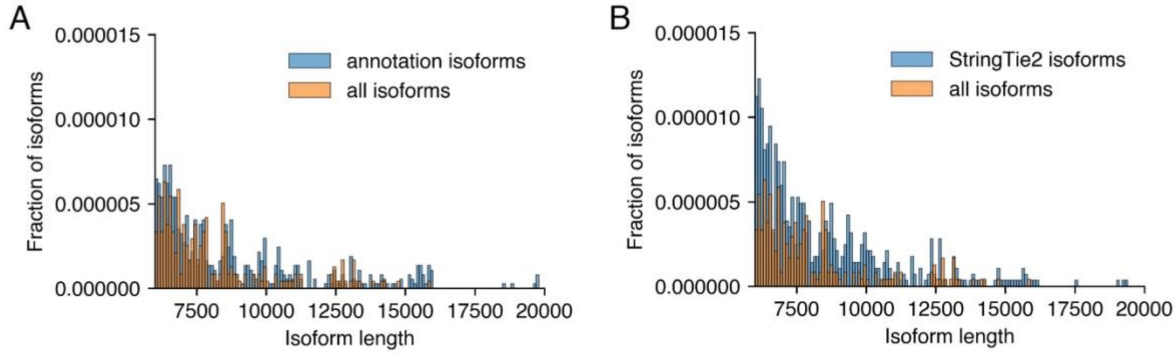

Supplemental Figure 3 – Histograms comparing isoform length densities at high lengths **(A)** Comparison of length distributions of isoforms present in the WormBase WS265 annotation, and splice isoforms identified by this study displayed as a density plot **(B)** As in A, comparison of length distribution of isoforms assembled by StringTie2 using Illumina based RNA-seq from across *C elegans* development, and splice isoforms identified by this study.

Supplemental Figure 4

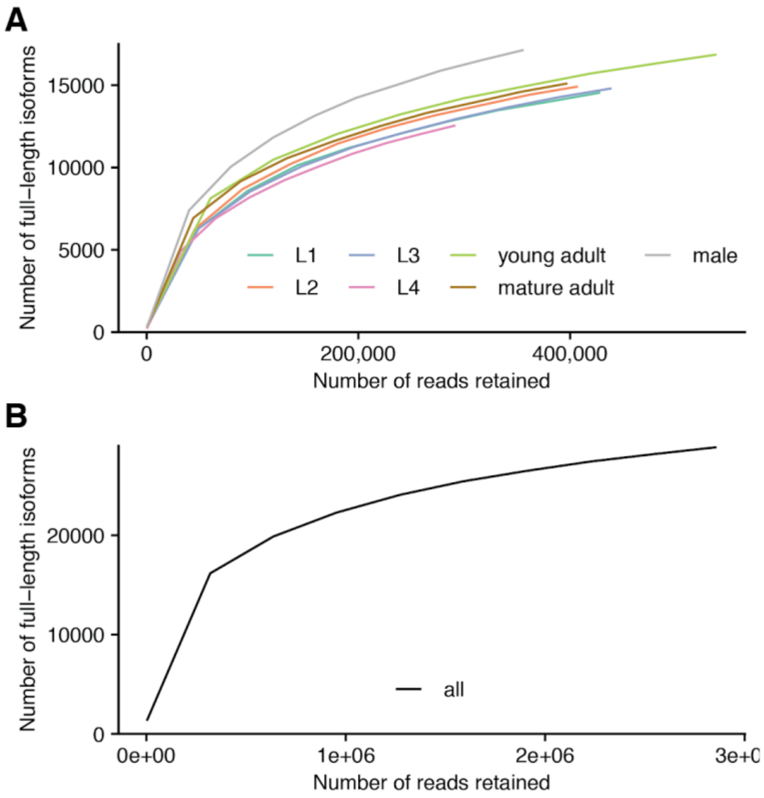

Supplemental Figure 4 - (A) Saturation plot showing the number of full-length isoforms with support from one or more reads versus the number of reads considered, separated by stage. **(B)** As in (A), but with all stages combined.

Supplemental Figure 5

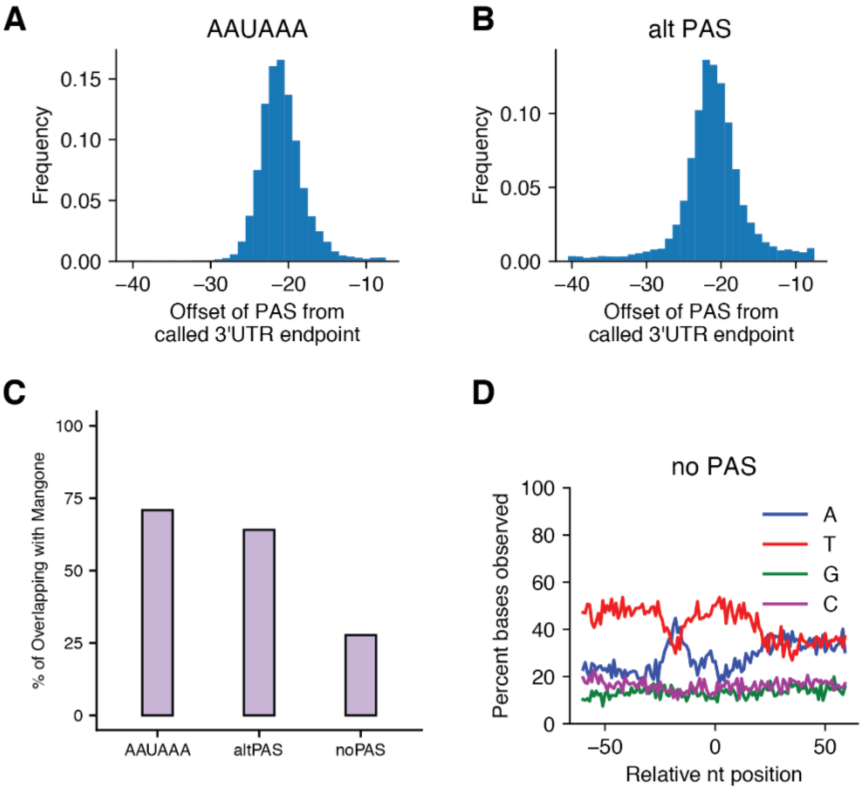

Supplemental Figure 5 - Evidence supporting the validity of our identified 3’UTRs. Offsets of identified PAS sites from the putative cleavage site for canonical **(A)** and non-canonical **(B)** PAS sites. **(C)** Percent of UTRs with specified PAS site type that overlap with a Mangone et al. 3’UTR. **(D)** Nucleotide distribution in a window around putative cleavage sites for 3’UTRs that overlap with a Mangone 3’UTR and do not have a PAS site identified. This distribution is different than the published distribution of no PAS Mangone 3’UTRs in general (Mangone et al. 2010)

Supplemental Figure 6

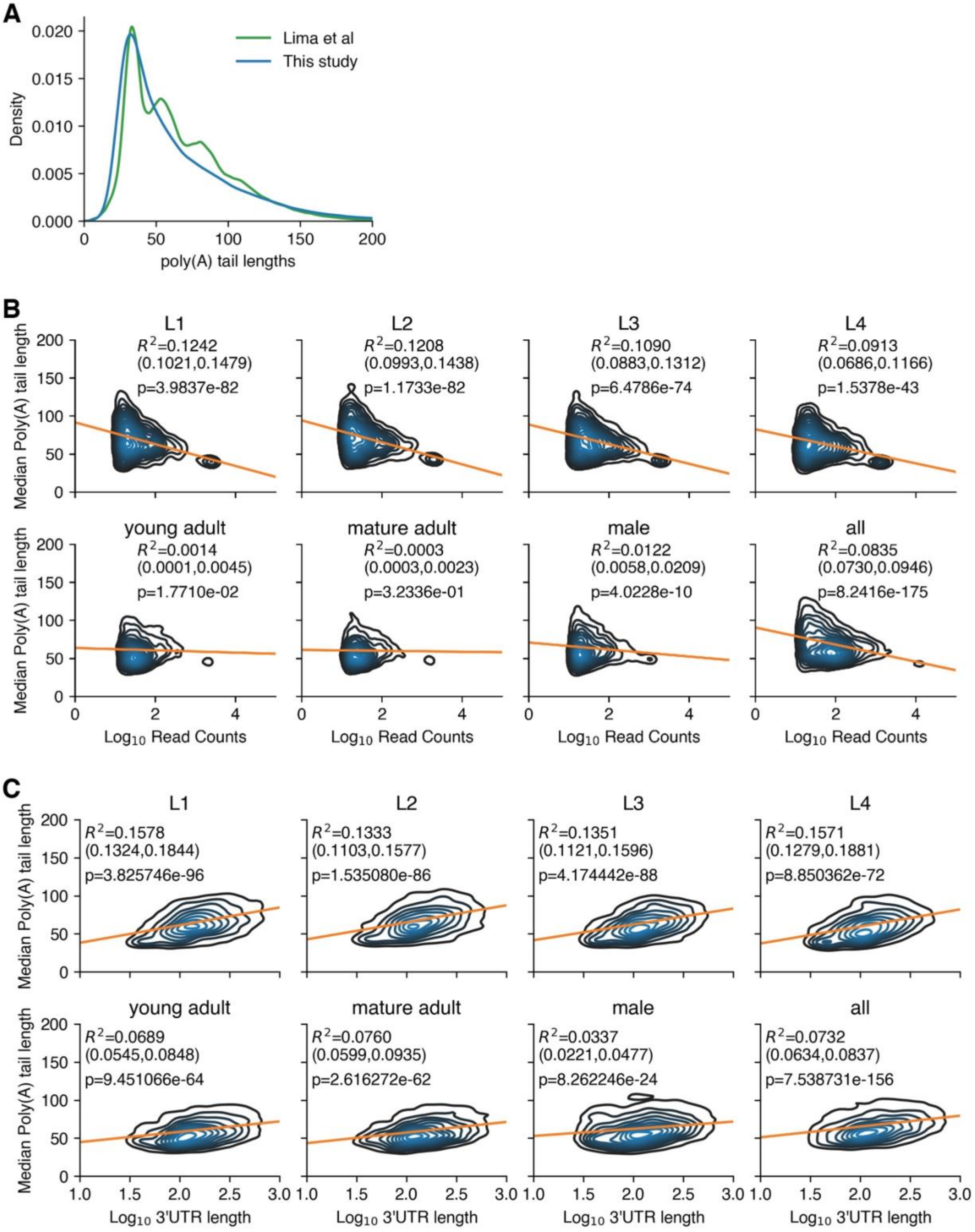

Supplemental Figure 6 - (A) Comparison of poly(A) tail length distributions between reads from our L4 stage dataset and Lima et al. (Lima et al. 2017). Density plots including linear regressions (orange line) of median poly(A) tail length versus expression level **(B)** or 3'UTR length **(C)**, separated by stage. Parenthesis indicate 95% confidence intervals for R^2 values. P-values calculated on Pearson correlation coefficients.
