# Supplementary material for: The full-length transcriptome of C. elegans using direct RNA sequencing
Source: Genome Res. 2020 Feb;30(2):299–312. doi: 10.1101/gr.251314.119 (PMC7050520; doi:10.1101/gr.251314.119)
Supplement: Supplemental Material [file supp_gr.251314.119_Supplemental_Code.tar.gz.zip › Supplemental_Code.tar.gz!/Supplemental_Code/results/trackHub/ce11_nanopore.html]

This is a track hub summarizing the results of a series of dRNAseq experiments of C. elegans RNA collected across development.

Details about the collection, sequencing, and analysis of these datasets can be found at (INSERT LINK HERE WHEN PAPER IS PUBLISHED).

A few disclaimers about these data:

- nanopore sequencing is not strictly quantitative, and so the relative ratios of genes / isoforms identified may not be representative of the true expression ratios.
- The throughput of dRNAseq is still quite limited, and these datasets are not saturated (i.e. more isoforms would be identified if we were to sequence more), and so if you do not see an isoform expressed in a given stage, that does not mean that the isoform is not expressed in that stage, it simply means we did not capture any reads corresponding to that isoform.
- 5' truncation of transcripts is quite common in dRNAseq data, and despite our various filtering steps, it is possible that we describe isoforms in our combinatorial full-length isoform track that are simply 5' truncations of known isoforms. Take any isoforms that are 5' truncations of known isoforms with a grain of salt as they may simply be artifacts of the known 5' truncation problem.
- Despite our best efforts, 3'UTRs identified in this dataset are approximations of the true cleavage site. Any given 3'UTR we identify may be several bp away from the true cleavage site, and our putative cleavage sites should not be treated as the exact cleavage site for any given 3'UTR.
